# Supplementary material for: The E3 ubiquitin ligase Pellino2 mediates priming of the NLRP3 inflammasome
Source: Nat Commun. 2018 Apr 19;9:1560. doi: 10.1038/s41467-018-03669-z (PMC5908787; doi:10.1038/s41467-018-03669-z)

The E3 ubiquitin ligase Pellino2 mediates priming of the NLRP3 inflammasome

Humphries *et. al.*

Supplementary Figures

Supplementary Figure 1

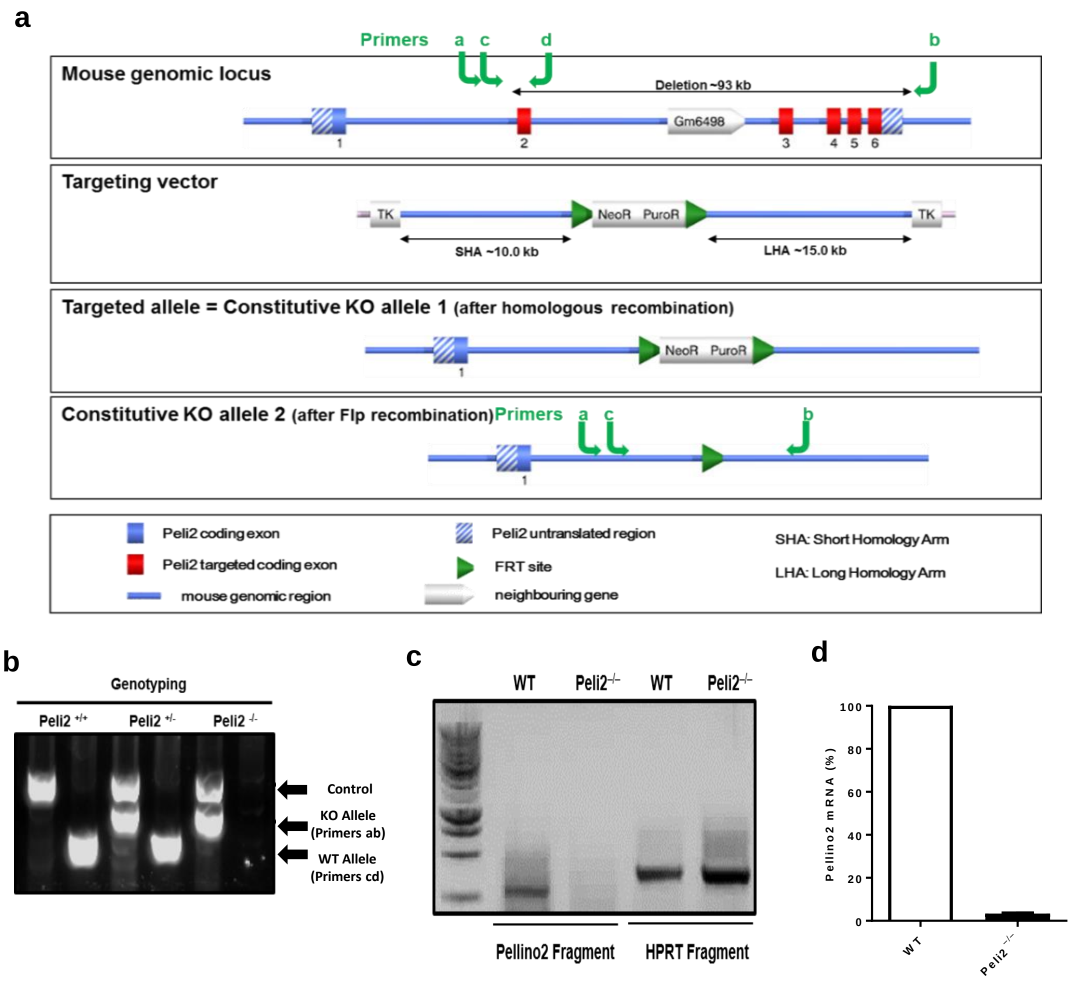

Supplementary Figure 1: Generation of Pellino-2 deficient mice. **(a)** Diagram shows the murine *Peli2* gene; the *Peli2* targeting vector containing the positive selection Puromycin cassette (PuroR) and a Thymidine Kinase (TK) gene; the targeted *Peli2* allele; and the *Peli2* knockout allele after Flp recombination. Homologous recombinants in ES cells were isolated by puromycin selection and used for blastocyst injection. Mice that were heterozygous for the targeted allele were bred with mice containing Flp recombinase, under the control of a chimeric CMV enhancer/β-actin promoter, generating constitutive Pellino2-deficient mice in which exons 2-6 have been deleted. Exons are numbered and regions targeted by genotyping primers are also indicated. **(b)** Genotyping by PCR analysis of genomic DNA from ear punches. Primers a and b differentiate the wild type (WT) allele from the *Peli2* knockout allele in heterozygous (hetero) and homozygous (homo) mice by amplifying a 341 base pair fragment from the knockout allele but not the WT allele. Primers c and d amplify a 208 base pair fragment from the WT allele but not the knockout allele. The integrity of PCR samples was confirmed by using primers to amplify a 585 base pair fragment from the CD79b wildtype allele (Control). **(c,d)** Semi-quantitative **(c)** and quantitative **(d)** RT-PCR analysis of *Peli2* mRNA expression in BMDMs from WT and *Peli2*$^{-/-}$ mice. Primers were designed to amplify a region of *Peli2* or a region of the housekeeping gene Hprt.

Supplementary Figure 2

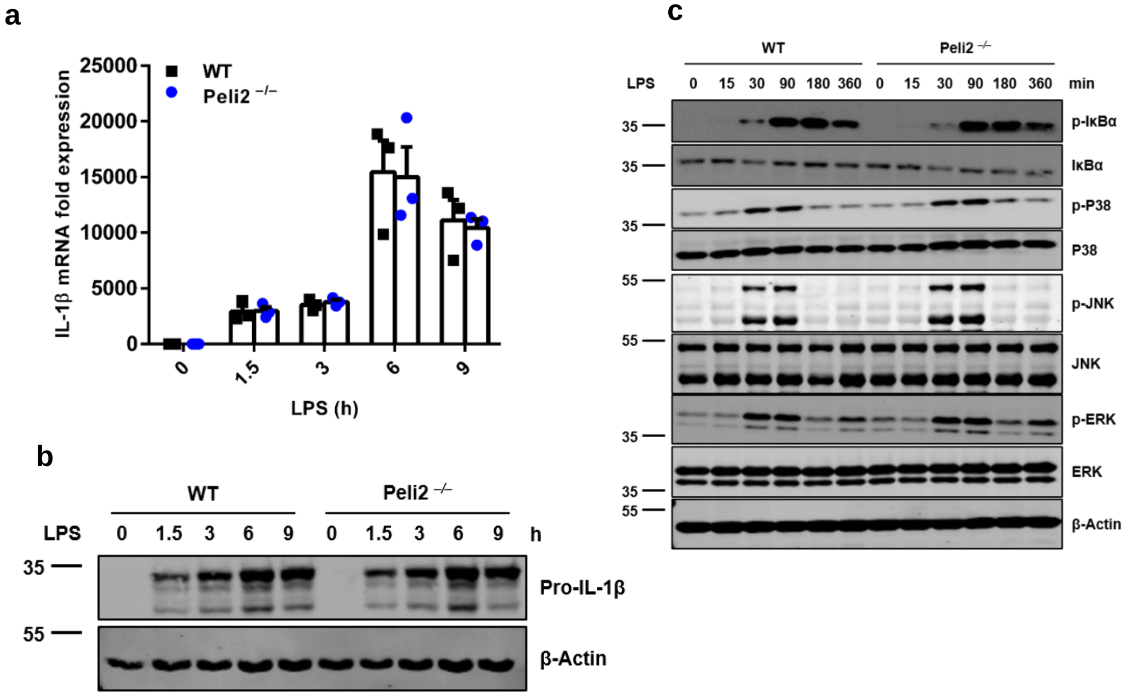

Supplementary Figure 2: Pellino2 does not mediate Signal 1 in NLRP3 pathway. (a) Quantitative RT-PCR analysis of *Il1β* mRNA expression in WT and *Peli2*$^{-/-}$ BMDMs treated with 100ng/ml LPS for the indicated times. (**b**) Immunoblot analysis of pro-IL-1β in cell lysates from WT and Peli2$^{-/-}$ BMDMs stimulated with 100 ng/ml LPS for the indicated times. (**c**) Immunoblot analysis of phosphorylated (p)-IκBα, total IκBα, p-P38, total P38 p-JNK, total JNK, p-ERK and total ERK in lysates from WT and *Peli2*$^{-/-}$ BMDMs stimulated with 100 ng/ml LPS for the indicated times. β-actin was used as loading controls. Data represent biological replicates and are representative of 3 experiments (**a**) or one experiment representative of three independent experiments (**b, d)**. Error bars, s.e.m.

Supplementary Figure 3

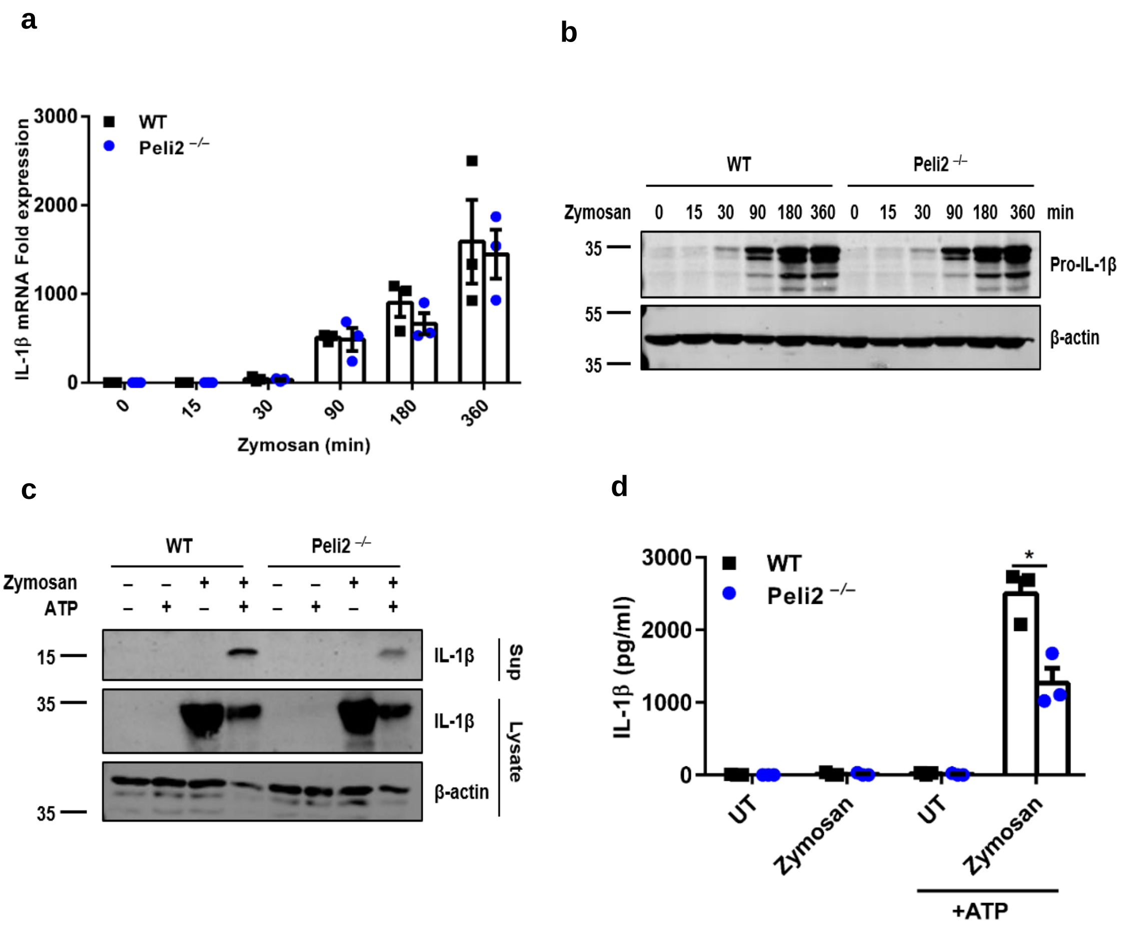

Supplementary Figure 3: Pellino2 mediates zymosan and ATP induction of mature IL-1β. **(a)** Quantitative RT-PCR analysis of *Il1β* mRNA expression in WT and *Peli2*$^{-/-}$ BMDMs treated with 100ng/ml zymosan for the indicated times. (**b**) Immunoblot analysis of pro-IL-1β in lysates from WT and *Peli2*$^{-/-}$ BMDMs stimulated with 100 ng/ml zymosan for the indicated times. β-actin was used as a loading control. (**c**) Immunoblot analysis of IL-1β in medium (sup) and cell lysates from WT and *Peli2*$^{-/-}$ BMDMs stimulated with 100 ng/ml zymosan for 3 h and 2.5mM ATP for 1 h. β-actin was used as a loading control. (**d**) ELISA of IL-1β in medium from WT and *Peli2*$^{-/-}$ BMDMs treated with 100ng/ml zymosan for 3 h and 2.5mM ATP for 1 h. *$P < 0.05$, paired Student's *t*-test. Data represent biological replicates and are representative of 3 experiments (**a,d**) or one experiment representative of three independent experiments (**b, c**). Error bars, s.e.m.

Supplementary Figure 4

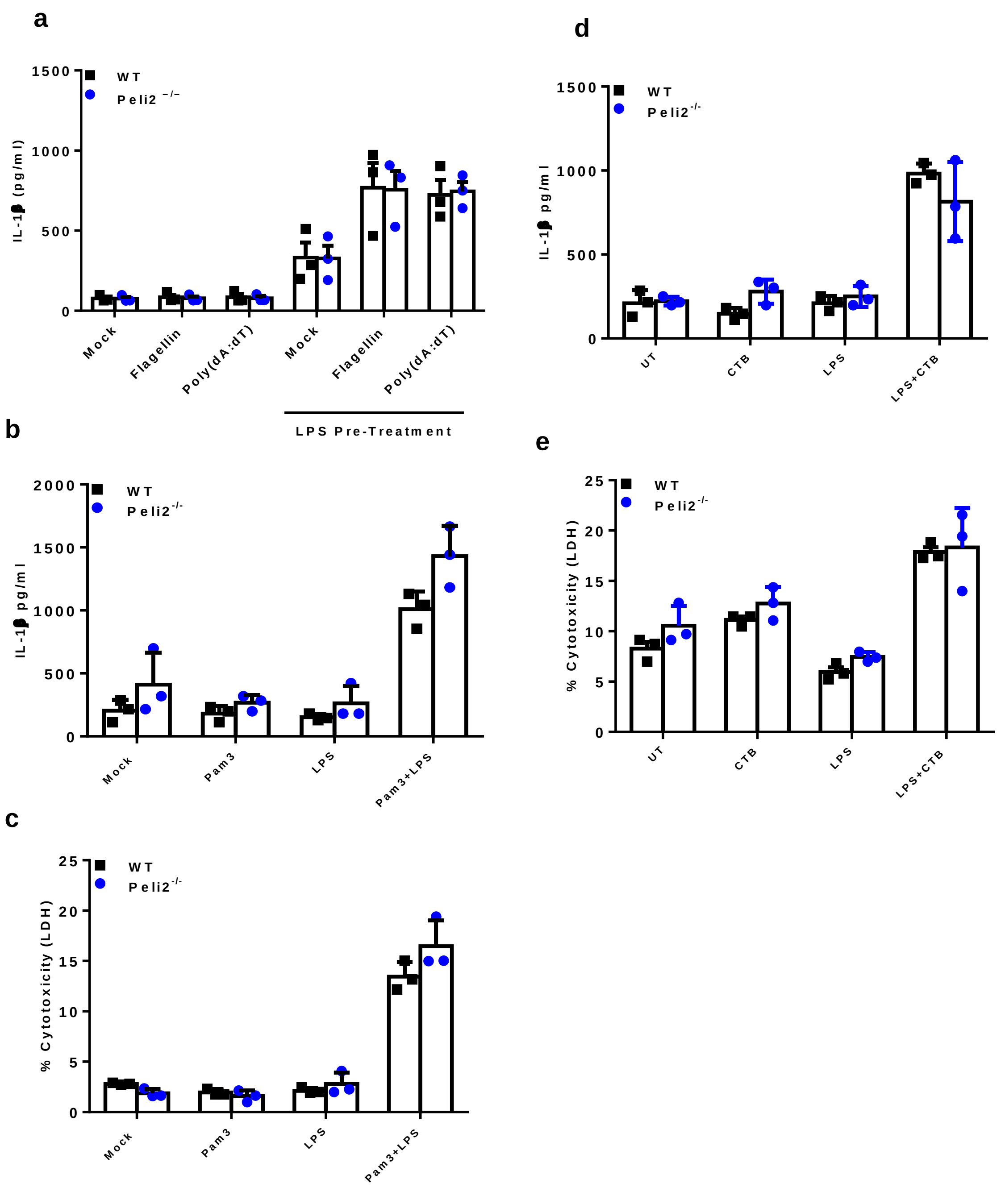

Supplementary Figure 4: Pellino2 does not mediate AIM2, NLRC4 or non-canonical inflammasome activation. (**a**) ELISA of IL-1β in medium from WT and *Peli2*$^{-/-}$ BMDMs treated with 100ng/ml LPS followed by transfection with 100ng/ml Poly(dA:dT) or 1μg/ml Flagellin for 16 h. (**b**) ELISA of IL-1β and (**c**) assay of LDH in medium from WT and *Peli2*$^{-/-}$ BMDMs treated with 1μg/ml Pam3CSK for 3 h followed by transfection of 1μg/ml LPS for 6 h. (**d**) ELISA of IL-1β and (**e**) assay of LDH in medium from WT and *Peli2*$^{-/-}$ BMDMs treated with 100ng/ml LPS for 3 h and Cholera Toxin B (CTB) 20μg/ml for 16 h. Data representative of 3 independent experiments presented as the mean +/- S.E.M.

Supplementary Figure 5

a

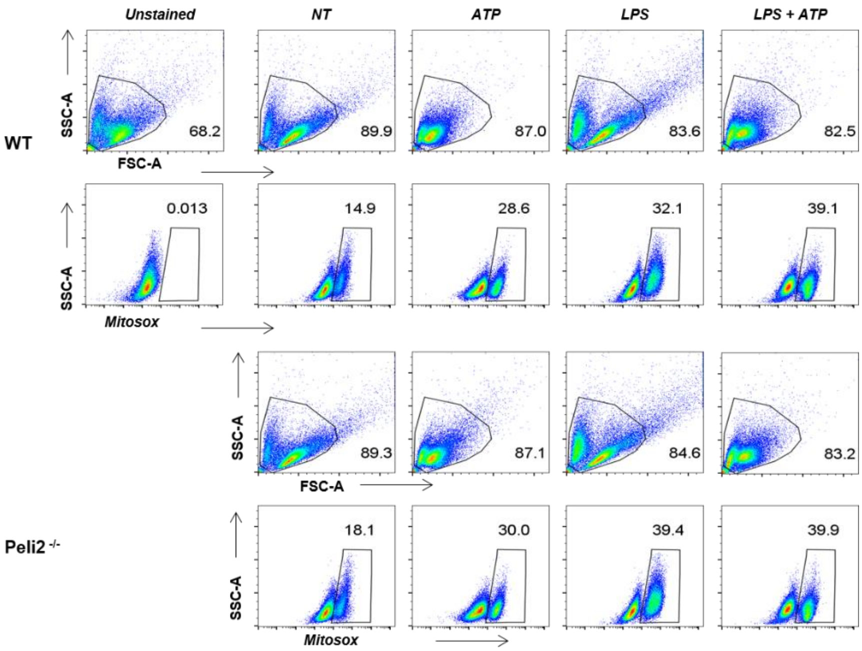

b

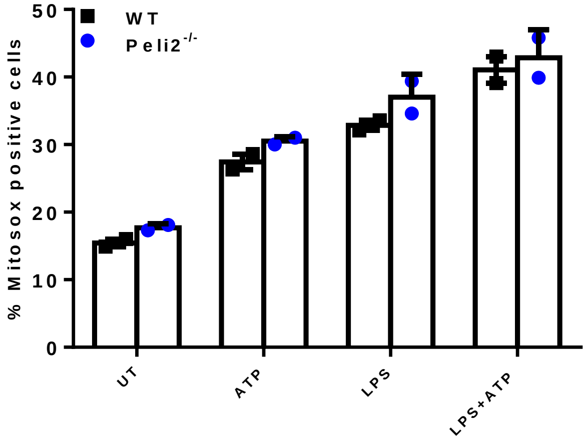

Supplementary Figure 5: Pellino2 does not mediate LPS/ATP induced production of mitochondrial reactive oxygen species. (**a**) Flow-cytometric analysis of Mitosox (5μM)-stained WT and *Peli2*$^{-/-}$ BMDMs treated with 100ng/ml LPS for 3 h with or without further stimulation with 2.5μM ATP for 30 min. (**b**) Histogram of percentage Mitosox positive cells. Data are presented as the mean +/- s.e.m of 2 experiments.

Supplementary Figure 6

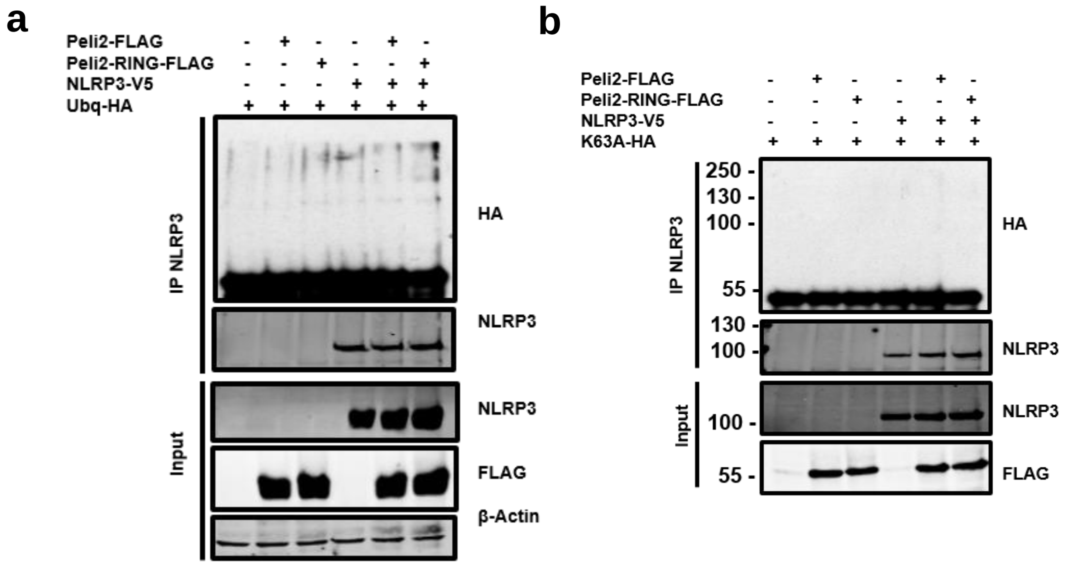

Supplementary Figure 6: Pellino2 is insufficient to ubiquitinate NLRP3. (**a, b**) Immunoblot analysis of HA, FLAG and NLRP3 in cell lysates (Input) and immunoprecipitated (IP) NLRP3 samples from HEK293T cells transfected with FLAG-tagged Pellino2, FLAG-tagged Pellino2 RING mutant, V5-tagged NLRP3 and (**a**) HA-Ubiquitin or (**b**) HA-K63A-ubiqutin. Data shown are representative immunoblots of 3 independent experiments.

Supplementary Figure 7

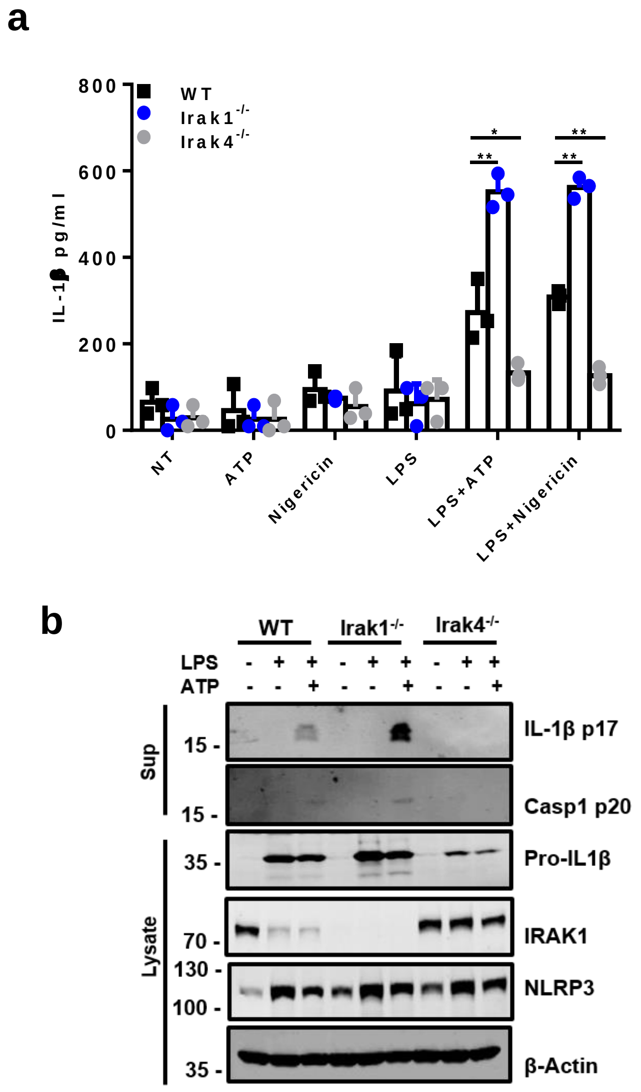

Supplementary Figure 7: IRAK1 suppresses IL-1β maturation in immortilised cells. (**a**) ELISA of IL-1β in medium from immortalized WT, *Irak1*$^{-/-}$ and *Irak4*$^{-/-}$ BMDMs treated with 100ng/ml LPS for 3 h and then with 2.5mM ATP or 5mM Nigericin for 1 h. (**b**) Immunoblot analysis of IL-1β and Caspase1 in medium (Sup) and lysates of immortalized WT, *Irak1*$^{-/-}$ and *Irak4*$^{-/-}$ BMDMs stimulated with 100 ng/ml LPS for 3 h and 2.5mM ATP for 1 h. Data are representative of 3 independent experiments with each experiment containing triplicate values (Error bars, s.d.).

Supplementary Figure 8: Uncropped blots relating to Figure 2

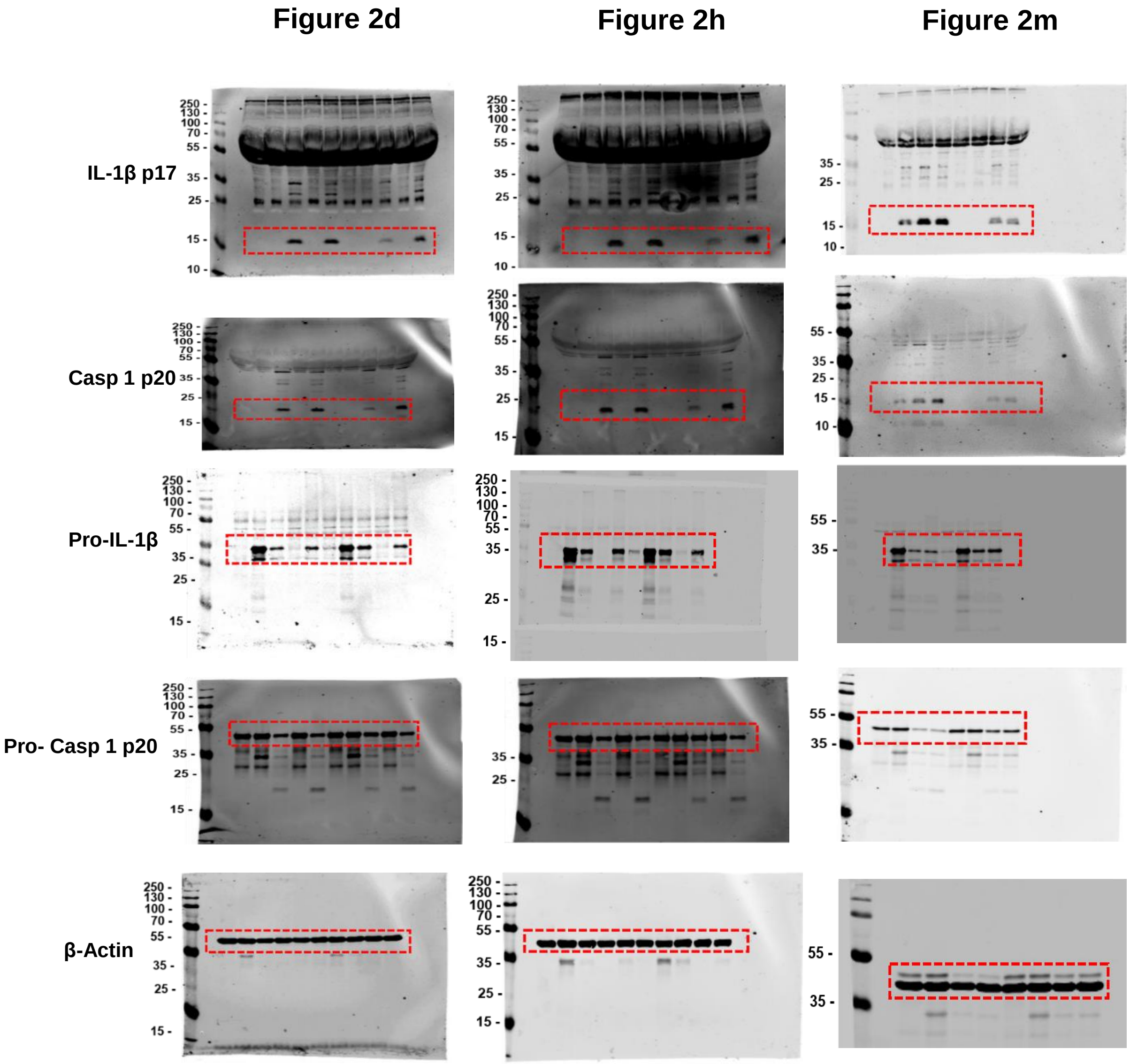

Supplementary Figure 9: Uncropped blots relating to Figure 4

Figure 4b

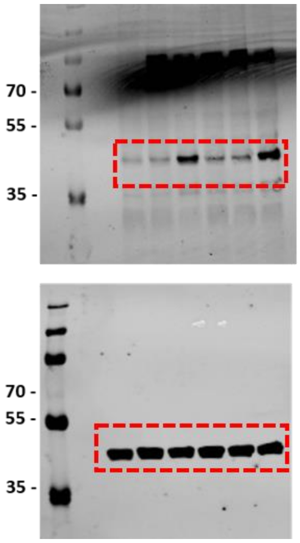

Figure 4d

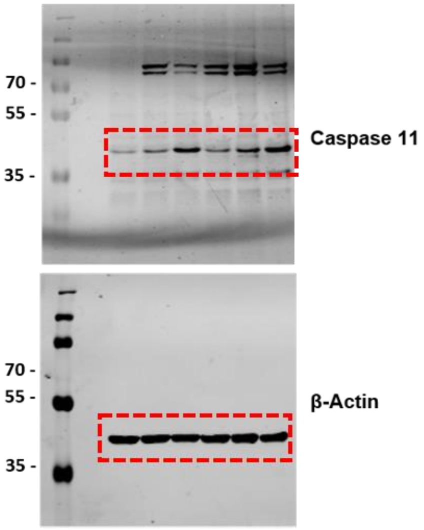

Figure 4f

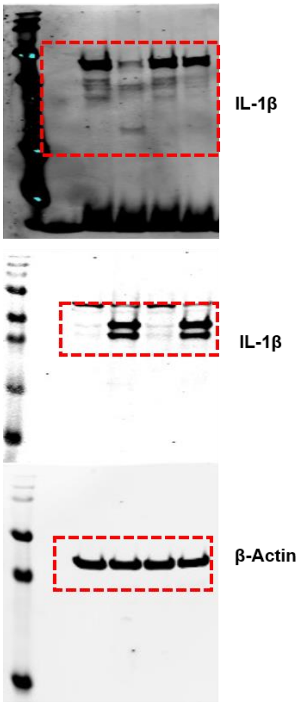

Supplementary Figure 10: Uncropped blots relating to Figure 5

Figure 5a

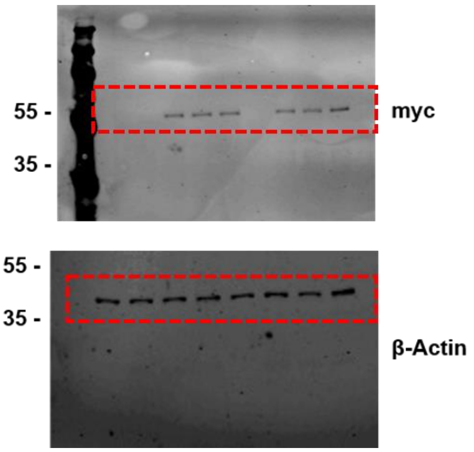

Figure 5c

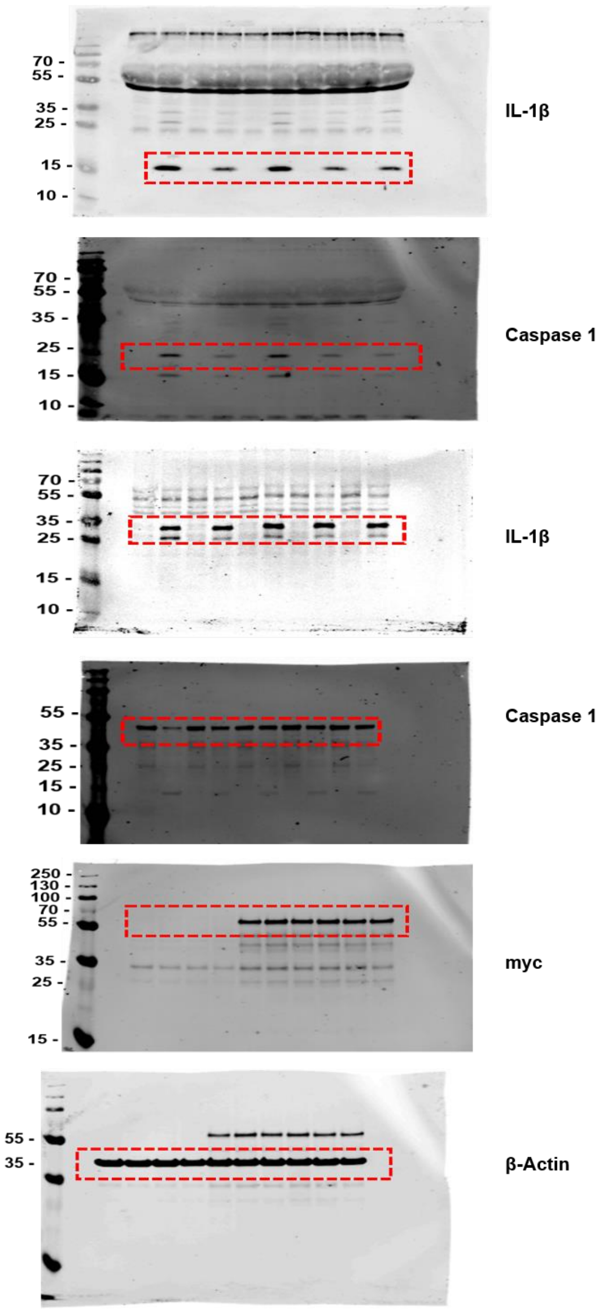

Supplementary Figure 11: Uncropped blots relating to Figure 6

Figure 6b

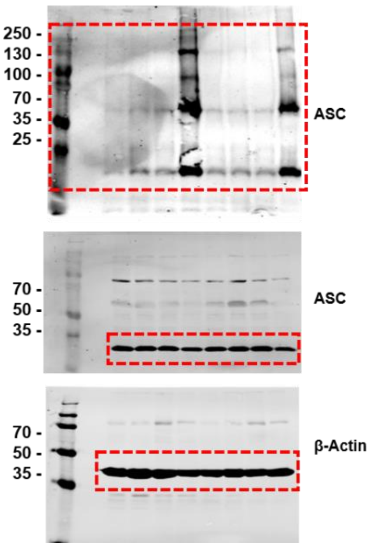

Figure 6c

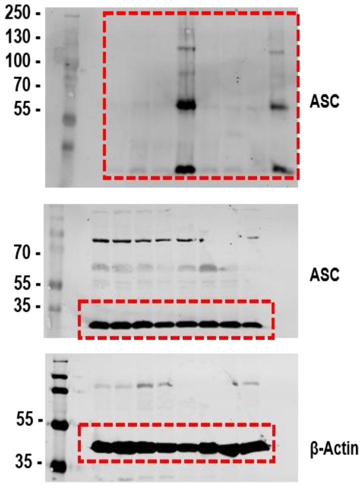

Figure 6d

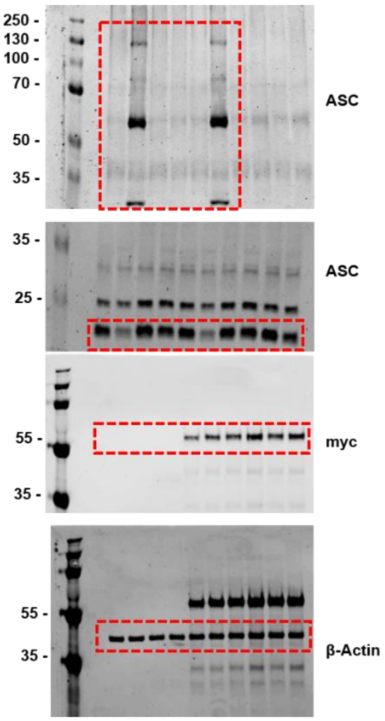

Supplementary Figure 12: Uncropped blots relating to Figure 7

Figure 7a

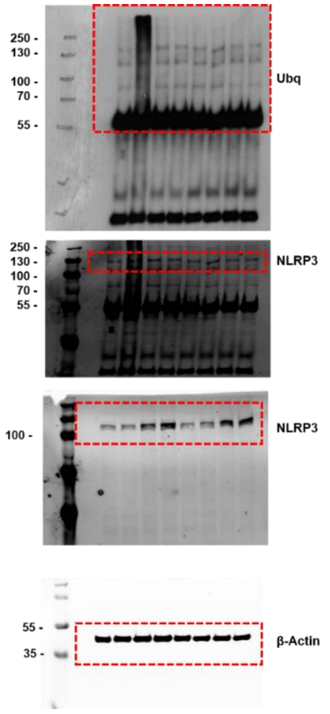

Figure 7b

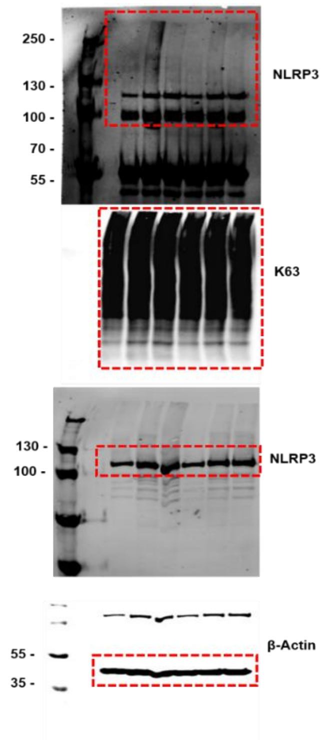

Figure 7c

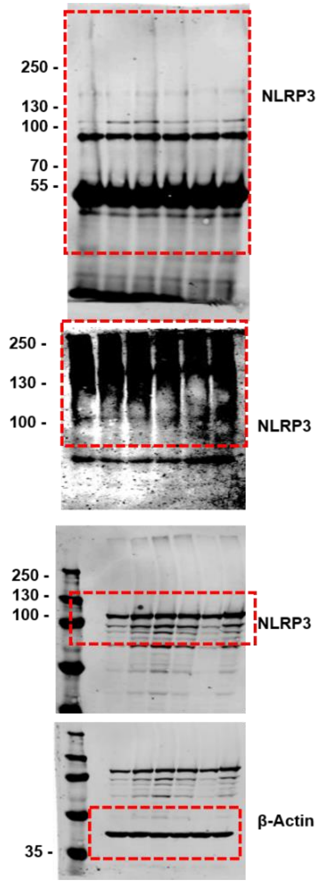

Figure 7e

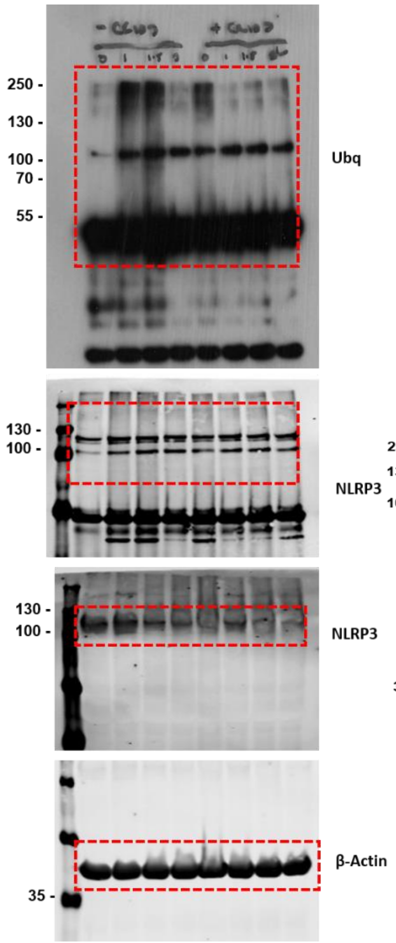

Figure 7f

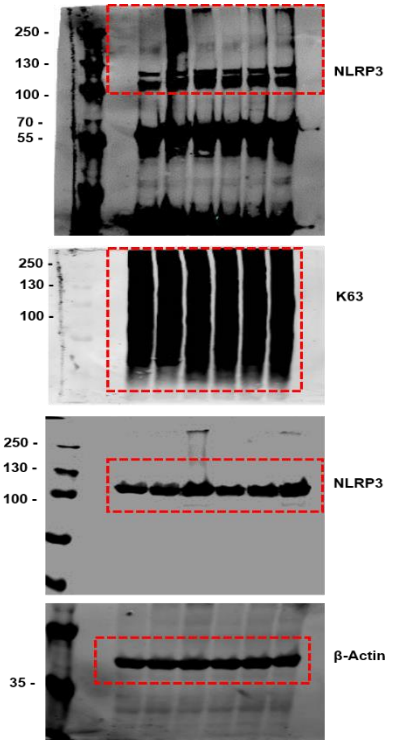

Figure 7g

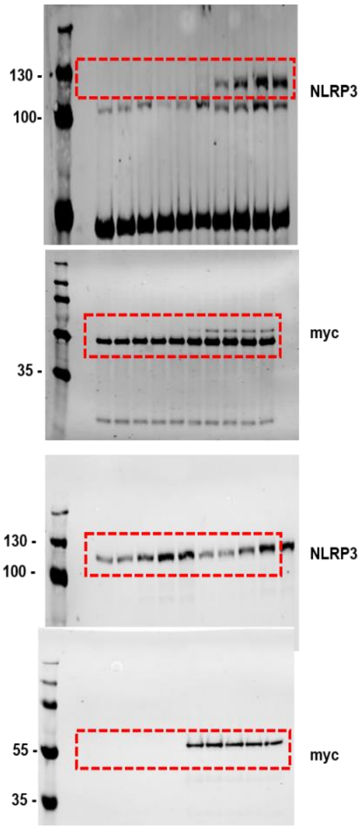

Supplementary Figure 13: Uncropped blots relating to Figure 8

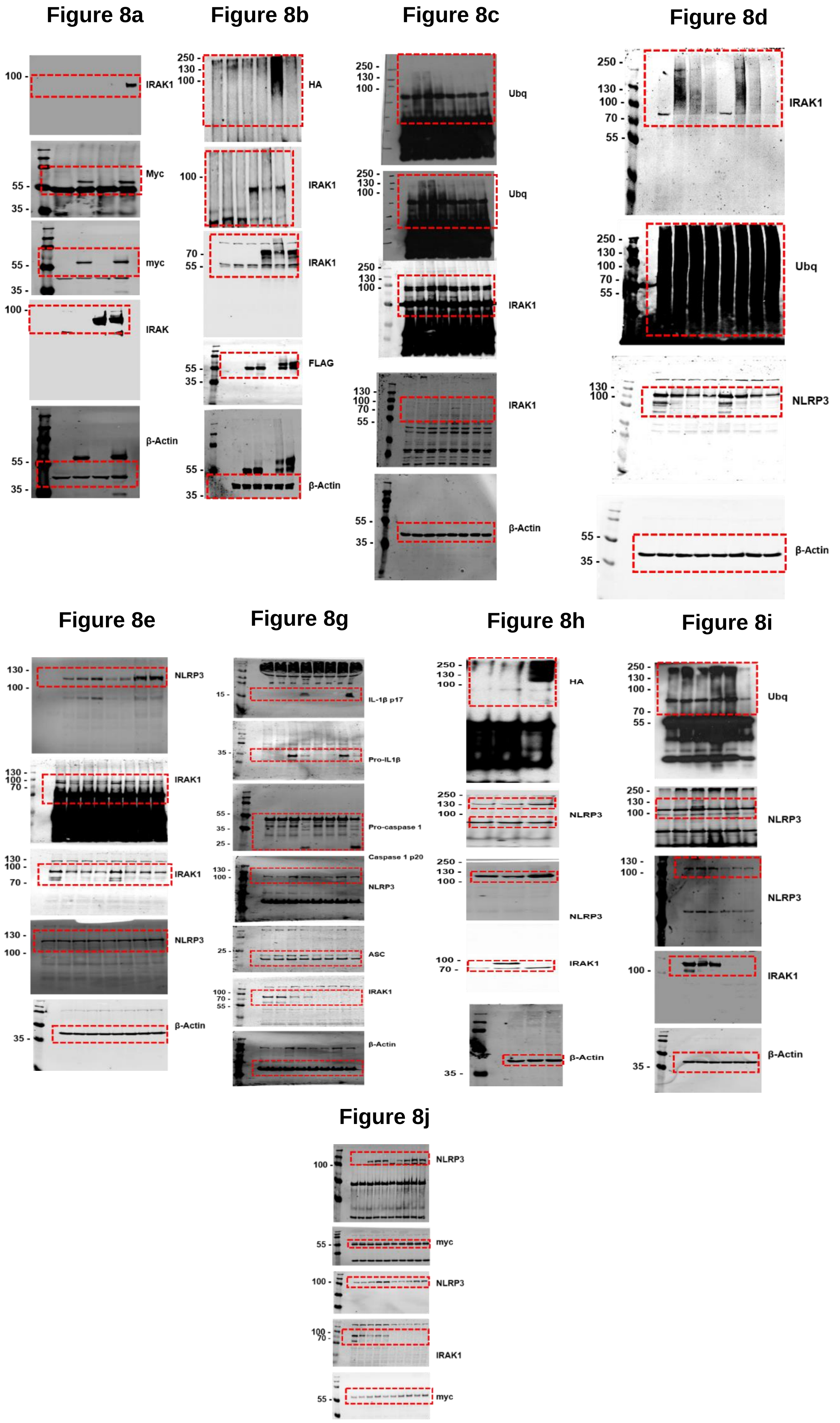

Supplementary Figure 14: Uncropped blots relating to Supp Figure 2

Supp Figure 2b

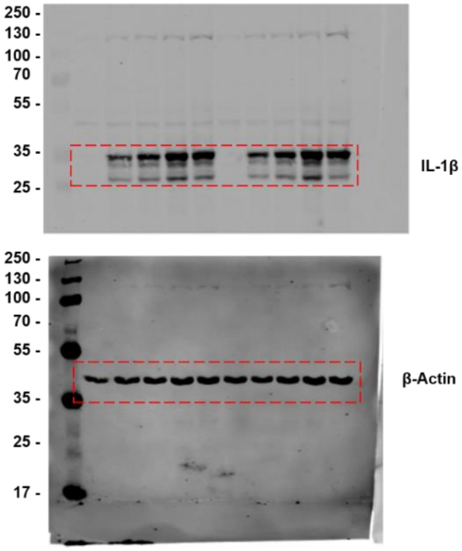

Supp Figure 2c

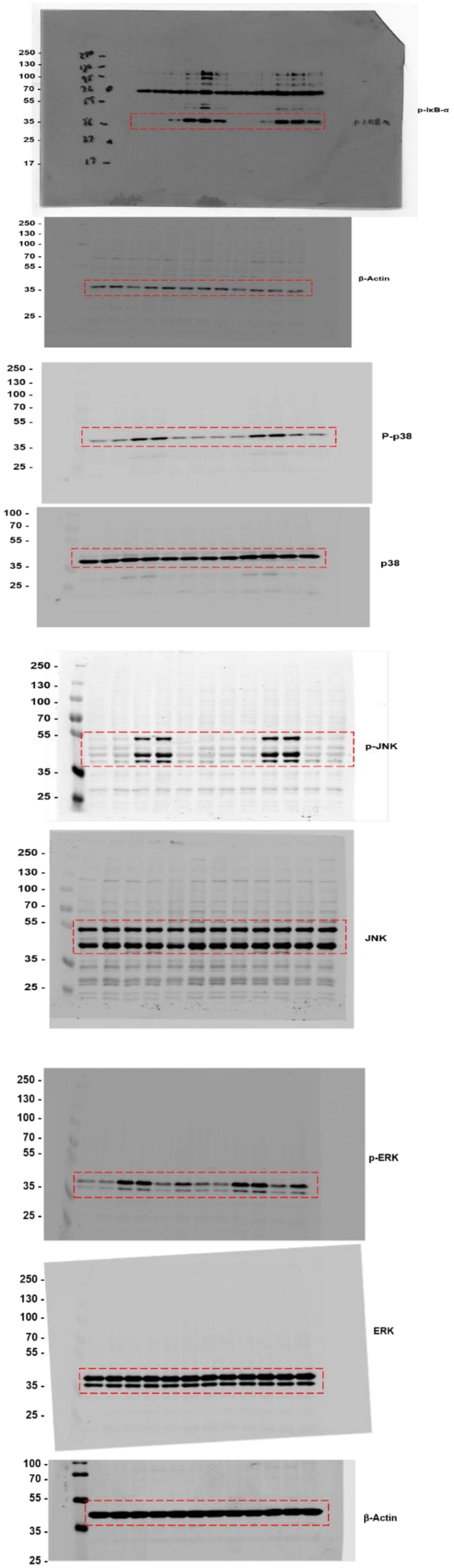

Supplementary Figure 15: Uncropped blots relating to Supp Figure 3

Supp Figure 3b

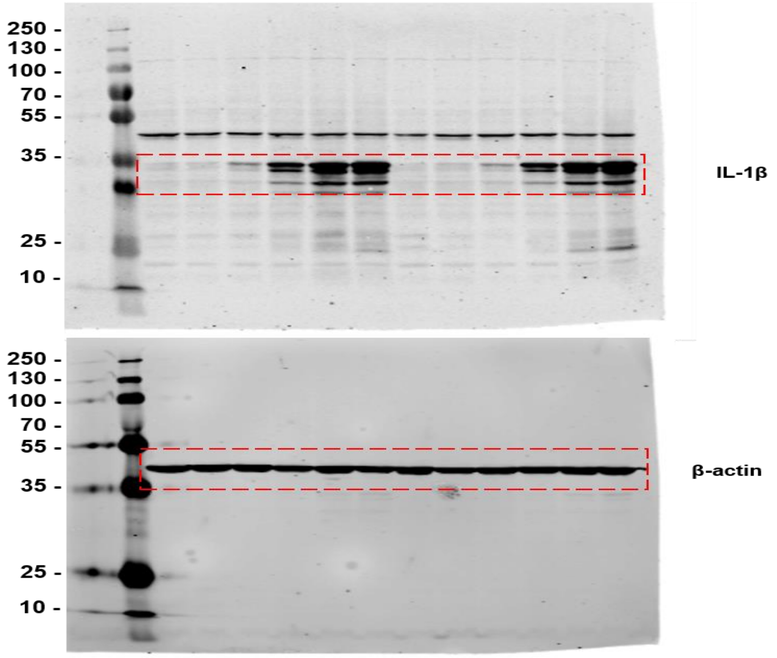

Supp Figure 3c

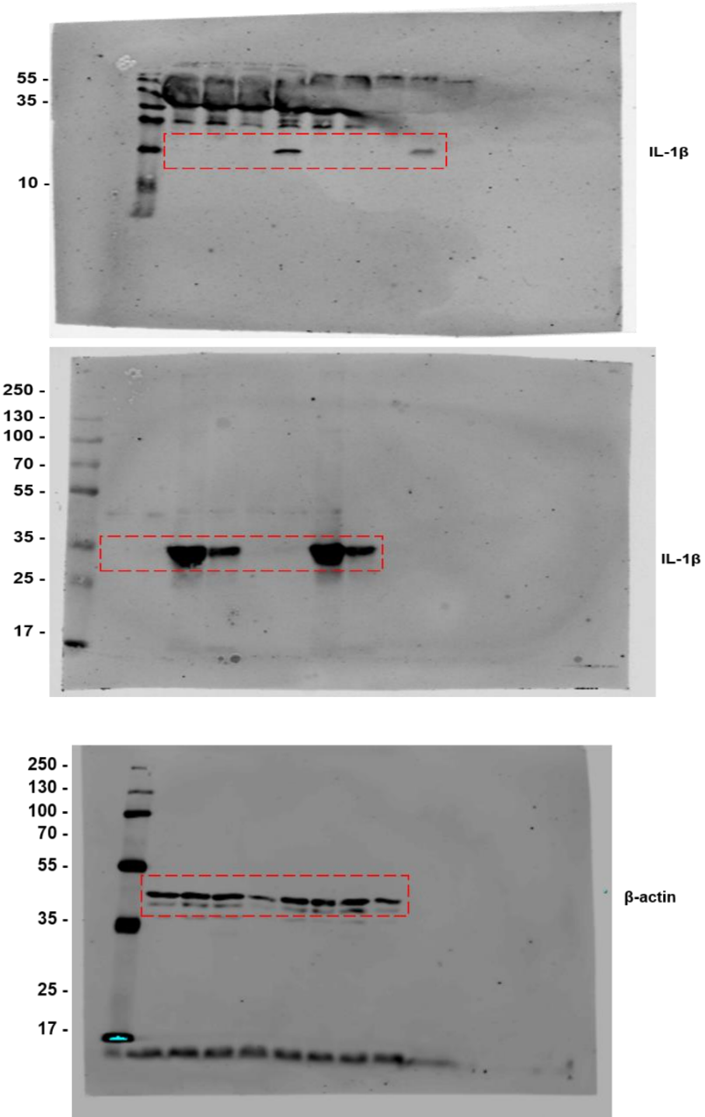

Supplementary Figure 16: Uncropped blots relating to Supp Figure 6

Supp Figure 6a

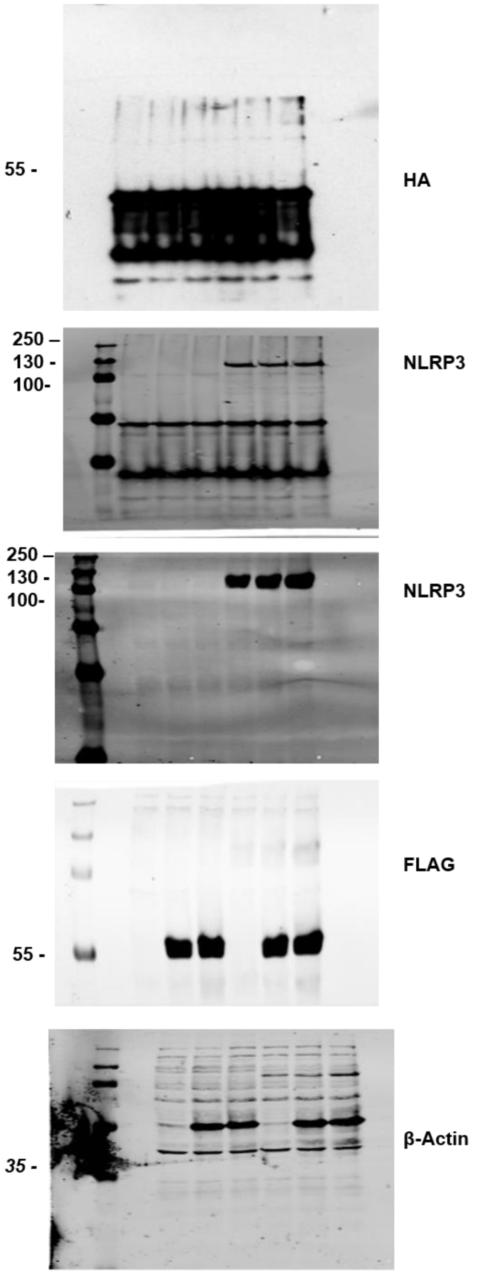

Supp Figure 6b

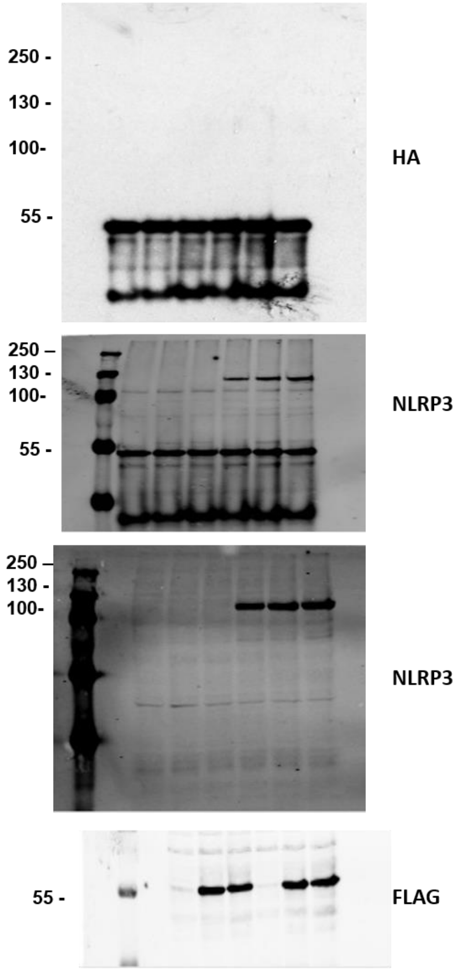

Supplementary Figure 17: Uncropped blots relating to Supp Figure 7

Supp Figure 7b

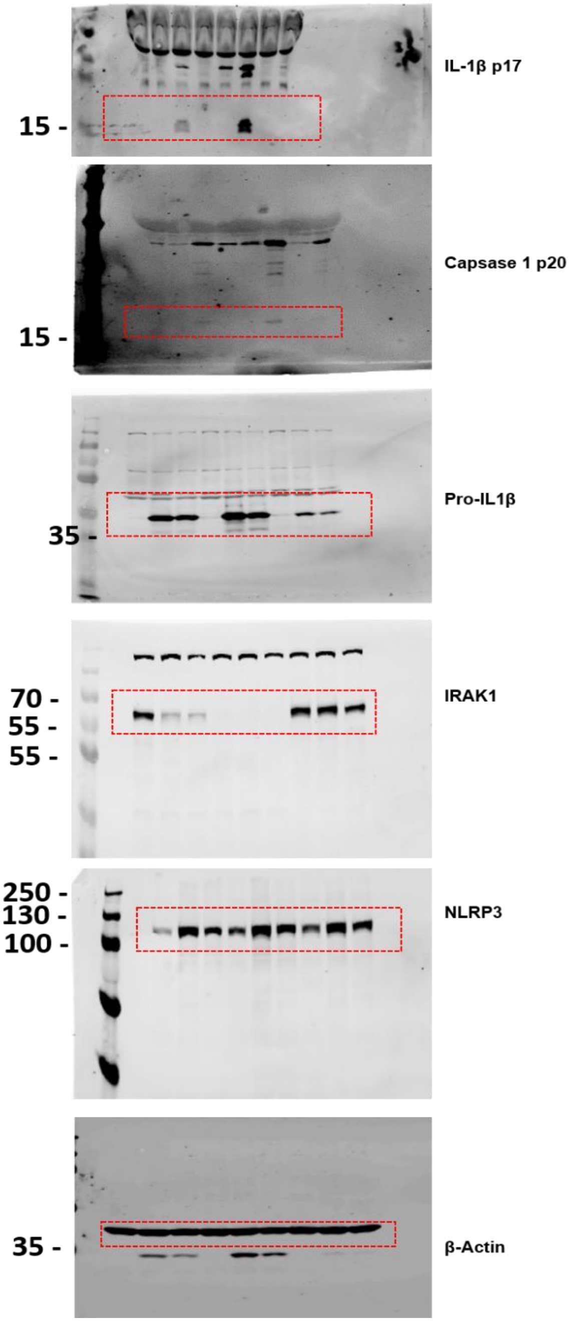

Supplement: Supplementary file 1 — Supplementary Information [file 41467_2018_3669_MOESM1_ESM.pdf]
